# HDAC inhibitors promote pancreatic stellate cell apoptosis and relieve pancreatic fibrosis by upregulating miR-15/16 in chronic pancreatitis

**DOI:** 10.1007/s13577-020-00387-x

**Published:** 2020-06-11

**Authors:** Ting Ji, Weiguang Feng, Xiangcheng Zhang, Kui Zang, Xingxing Zhu, Futai Shang

**Affiliations:** 1 Intensive Care Unit, The Affiliated Huai’an No. 1 People’s Hospital of Nanjing Medical University, Beijing West Road, Huaiyin District, Huai’an, 223300 Jiangsu China; 2 Intensive Care Unit, Huai’an No 4 People’s Hospital, 128 Yan’an East Road, Qingjiangpu District, Huai’an, 223002 Jiangsu China

**Keywords:** Chronic pancreatitis, HDAC inhibition, Apoptosis, miR-15/miR-16

## Abstract

In chronic pancreatitis, PSCs are activated by proinflammatory cytokines to induce pancreatic fibrogenesis. HDAC inhibition protected against the pancreatic fibrosis and the apoptosis of PSCs through induced apoptosis and depressed inflammation. In our study, we found that miR-15 and miR-16 decreased significantly in chronic pancreatitis and HDAC inhibition could recover the levels of these two miRNAs. HDAC regulated the transcription of miR-15 and miR-16, which then modulate the apoptosis and fibrosis of PSCs. And we proved that Bcl-2 and Smad5 were the target genes of miR-15 and miR-16, which illustrated how HDAC inhibition alleviated the apoptosis and fibrogenesis of PSCs in chronic pancreatitis. These results suggested that HDAC inhibition protects against CP by promoting apoptosis and TGF-β/Smads signaling pathways, and indicated that HDAC inhibition is a potential therapy to alleviate CP patients in clinic, and these need to be explored further.

**Electronic supplementary material:**

The online version of this article (10.1007/s13577-020-00387-x) contains supplementary material, which is available to authorized users.

## Introduction

Chronic pancreatitis (CP) usually came from long-term damage to the pancreas, eventually caused endocrine and exocrine deficiency characterized by maldigestion and diabetes [1, 2]. The histologic features of CP include chronic inflammatory, fibrosis, acinar cell atrophy and distorted or blocked ducts [2–4]. Once damaged, pancreatic stellate cells (PSCs) are activated by proinflammatory cytokines to induce pancreatic fibrogenesis [5]. They can secrete excessive amounts of extracellular matrix (ECM) proteins which comprise fibrous tissue [6, 7]. Many researchers have attempted to identify how inflammation and apoptosis of the PSCs caused chronic pancreatitis eventually, but the details of the mechanism were still unknown.

HDAC enzymes involve removing the acetyl group from the histones comprising the nucleosome, and decreased levels of acetylation are usually associated with repression of gene expression [8, 9]. In recent years, HDAC inhibitors have emerged as a novel class of agents that regulate chromatin structure and gene expression, inducing growth inhibition, apoptosis, and differentiation, such as Vorinostat (SAHA) and Trichostatin A (TAS) [10–12].

TGF-β is a special growth factor and at the same time is chemotactic for fibroblasts, stimulates fibroblast proliferation, and increases the synthesis of a number of extracellular matrix proteins [13, 14]. It promotes the initiation and progression of pancreatic fibrosis through matrix production and growth control of the pancreatic stellate cells in vitro [15–17]. The Smads are a family of intracellular regulatory proteins that act downstream of the TGF-β Type I receptor. Once activated, Smad complex could translocate to the nucleus where it is recruited to DNA to regulate the transcription of specific genes [18, 19]. Smad7, as one of the inhibitory Smads, which antagonize the activity of the receptor-regulated Smads [20, 21]. Abnormal Smad signaling is likely to be related to the onset and progression of fibrosis [22–24].

It is well known that miRNAs are involved in a variety of biological processes, including cell proliferation, differentiation, apoptosis, and inflammation [25–27]. Abnormal expression of miRNAs is often associated with pathological disorders [28, 29]. Therefore, the identification of abnormally expressed miRNAs will be helpful in further understanding of CP [30, 31]. Some study has reported that miR-15 and miR-16 may regulate the expression of Bcl-2, an important apoptosis-related protein [32]. Moreover, It has been learned that miR-15a/miR-16 cluster suppressed TGF-β signaling pathway through Smads expression in tumor invasion [33]. Therefore, we focused our further study on miR-15 and miR-16, explored the relationship between them and the apoptosis and fibrosis of activated PSCs in vitro.

In this study, we found that HDAC inhibition could rescue the pancreatic fibrosis in chronic pancreatitis through increased expression of miR-15 and miR-16. The abnormal surplus miR-15 and miR-16 in pancreatic tissues from chronic pancreatitis restored after HDAC inhibition. And related pathological damage such as fibrosis and inflammation were alleviated as well. The apoptosis of PSCs was promoted after either overexpression of miR-15 or miR-16 or HDAC inhibition, but TGF-β/Smads signaling pathway were repressed at the same time. According to our research, HDAC inhibition could induce the transcription of miR-15 and miR-16, additionally both Bcl-2 and Smad5 were the target genes of miR-15 and miR-16. These results suggested that HDAC inhibition protected against the pancreatic fibrosis and the apoptosis of PSCs through induced apoptosis and depressed inflammation by increased miR-15 and miR-16. So HDAC inhibitors are potential therapies to treat CP patients in the future.

## Materials and methods

### Animals and induction of CP

Male Sprague–Dawley rats used in this study were purchased from Slac Laboratory Animal Company (Shanghai, China). Animals were maintained on a 12 h light/12 h dark cycle at 22 °C, given ad libitum access to food and water. All experiments were conducted with the approval of the Animal Research Committee at Changzhou City No. 1 People’s Hospital. Chronic pancreatitis model was induced as previously described [34]. Briefly, food was withdrawn 12 h prior to induction, the common bile duct was closed temporarily near the liver with a small vascular clamp. A blunt 24-gauge needle was inserted into the duodenum and guided through the papilla into the duct and was secured with suture. 0.5 ml TNBS (trinitrobenzene sulfonic acid) solution (2%) in 10% ethanol in PBS was infused into the pancreatic duct. After 30 min, needle and suture line were removed, the hole in the duodenum was sutured, whereas, in the control group, saline was infused instead of TNBS.

### Reagents and drugs

Vorinostat (SAHA) and Trichostatin A (TSA) were purchased from MedChem Express (Shanghai, China). Anti-GFAP (sc-33673) was purchased from Santa Cruz (Dallas, TX, USA). Anti-α-SMA (A5228), Anti-Desmin (D1033), Anti-acetyl-Histone H3 (H9286), Anti-acetyl-Histone H4 (SAB5600021) and Anti-actin (A5060) were purchased from Sigma-Aldrich (St. Louis, MO, USA). Anti-Cleaved Caspase-9 (9507), Anti-Cleaved Caspase-3 (5A1E), Anti-Smad5 (12534) were purchased from Cell Signaling (Beverly, MA, USA). Anti-Smad7 (42-0400) and Bcl-2 (13-8800) were purchased from Invitrogen (Carlsbad, CA, USA). RT reagent kit and Premix Ex Taq were from TAKARA (Dalian, China). Lipofectamine 2000 was purchased from Invitrogen (Carlsbad, CA, USA). Luciferase kit was purchased from Promega (WI, USA). Apoptosis kit was purchased from BD Biosciences (San Jose, CA, USA).

### Isolation, identification and culture of pancreatic stellate cells

Primary pancreatic stellate cells were isolated from normal male Sprague–Dawley rats by digestion of pancreatic tissue and Nycodenz density gradient centrifugation [35]. They were cultured at 37° C in a 5% CO2. The medium consisted of Dulbecco’s modified Eagle’s medium/Ham F12 medium (1:1, vol/vol) with 10% FBS (Gibco, 10091155, Waltham, Ma, USA), 100 U/ml penicillin and 100 mg/ml streptomycin. Primary pancreatic stellate cells cultured over 3 generations were used for the experiments.

### ELISA

Serum concentrations of TNF-α and IL-6 were determined via ELISA assay kits (R&D, Minneapolis, MN, USA).

### Stem-loop RT-PCR to measure micro RNA levels

The samples of pancreatic tissues and primary pancreatic stellate cells were collected with TRIzol reagent (Invitrogen, Carlsbad, CA, USA) to achieve total RNA. Expression of mature miR-15 and miR-16 were assayed using stem-loop RT followed by PCR analysis as previously described [36]. The relative amount of miRNA was normalized to U6 snRNA. The RT primer for miR-15 was GTCGTATCCAGTGCAGGGTCCGAGGTATTCGCACTGGATACGACTGTAAA. The RT primers for miR-16 was GTCGTATCCAGTGCAGGGTCCGAGGTATTCGCACTGGATACGACCGCCAA. The RT primers for U6 was CGTTCACGAATTTGCGTGTCAT. The qPCR primers for miR-15 were GGCGGTAGCAGCACATCATG (forward primer) and GTGCAGGGTCCGAGGT (reverse primer). The qPCR primers for miR-16 were GGCGGTAGCAGCACGTAAATA (forward primer) and GTGCAGGGTCCGAGGT (reverse primer). The qPCR primers for U6 were GCTTCGGCAGCACATATACTAAAAT (forward primer) and CGCTTCACGAATTTGCGTGTCAT (reverse primer).

### RT and qPCR

The samples of pancreatic tissues and primary pancreatic stellate cells were collected with TRIzol reagent (Invitrogen, Carlsbad, CA, USA) to achieve total RNA. 0.5 μg of total RNA was reverse-transcribed to synthesize cDNA using a first-strand cDNA synthesis kit (TAKARA, Dalian, China). qPCR was performed through the ABI PRISM 7500 Fast Sequence Detection System (Applied Biosystems, USA) using the SYBR Green PCR kit (Applied Biosystems, USA). Relative mRNA levels were normalized to GAPDH mRNA, and the fold change for each mRNA was calculated using the ∆∆Ct method. The mRNA primers for α-SMA were TTCCAGCCTTCCTTTATCG (forward primer) and TTGGCGTACAGGTCCTTC (reverse primer). The mRNA primers for GAPDH were TATCGGACGCCTGGTTAC (forward primer) and CTGTGCCGTTGAACTTGC (reverse primer).

### Western blot

Related primary pancreatic stellate cells were harvested by RIPA lysis buffer containing 1 mmol/l PMSF and centrifuged at 12000 rpm for 10 min at 4 °C. All protein sample concentration was determined by the BCA method (Thermo, Carlsbad, CA, USA). The proteins were separated by 10% or 15% of SDS/polyacrylamide gels, which were transferred to the PVDF membranes (Bio-Rad, Hercules, CA, USA). The membranes were blocked in 5% milk for 1 h at RT, then incubated with primary antibodies overnight at 4 °C. The next day, the membranes were washed with TBST for three times, incubated with HRP-labeled secondary antibodies (Cell Signaling, Beverly, MA, USA). The ECL reagents (Thermo, Carlsbad, CA, USA)were added to visualize the chemiluminescence by ECL Plus detection system (Tanon, Shanghai, China). The band densities were analyzed with the ImageJ analysis system.

### Immunohistochemistry and immunofluorescence staining

The pancreatic tissues were fixed in 10% formalin, paraffin embedded and mounted to sections. Primary pancreatic stellate cells were seeded in 6-well plates with 5 × 104 cells/well. Cells were transfected with miR-15 and miR-16 mimics. After 48 h, they were fixed with neutral formalin. Both tissues and cells were stained with α-SMA (dilution 1:200), and the nucleus was stained with propidium iodide (PI). The images were captured by fluorescence microscope.

### Apoptosis analysis

CCK-8 reagent was purchased from Dojindo laboratories. To measure the double-stranded cleavage of DNA, TUNEL assay was performed with an in-situ cell death detection kit following the manufacturer’s instructions (R&D, Minneapolis, MN, USA). The Annexin-V-FITC/propidium iodide (PI) Apoptosis Detection Kit (BD, San Jose, CA, USA) was used to evaluate cell apoptosis according to the manufacturer’s instruction. TUNEL Assay and Apoptosis Assay. The samples were detected by FACS (BD, San Jose, CA, USA). The experiments were repeated three times.

### Caspase-3, caspase-9 activity assay

Primary pancreatic stellate cells were seeded in six-well plates and transfected with miR-15, miR-16, miR-15 inhibitor or miR-16 inhibitor with the company of SAHA. Caspase-3 and caspase-9 activities were detected using commercially available kits (KGA204 and KGA404) from KeyGen BioTech (Jiangsu, China) according to the manufacturer’s instructions. Caspase activity was expressed as a ratio of the absorbance of control cells.

### Statistical analysis

Results were presented as mean ± SD, and statistical analysis was performed using Prism GraphPad. Unpaired Student’s *t* test was used to determine statistical significance unless otherwise indicated, and *P* value of less than 0.05 was considered significant. *** means *P* < 0.001.

## Results

### Vorinostat (SAHA) prevented pancreatic fibrosis in rats of chronic pancreatitis

**Fig. 1:**
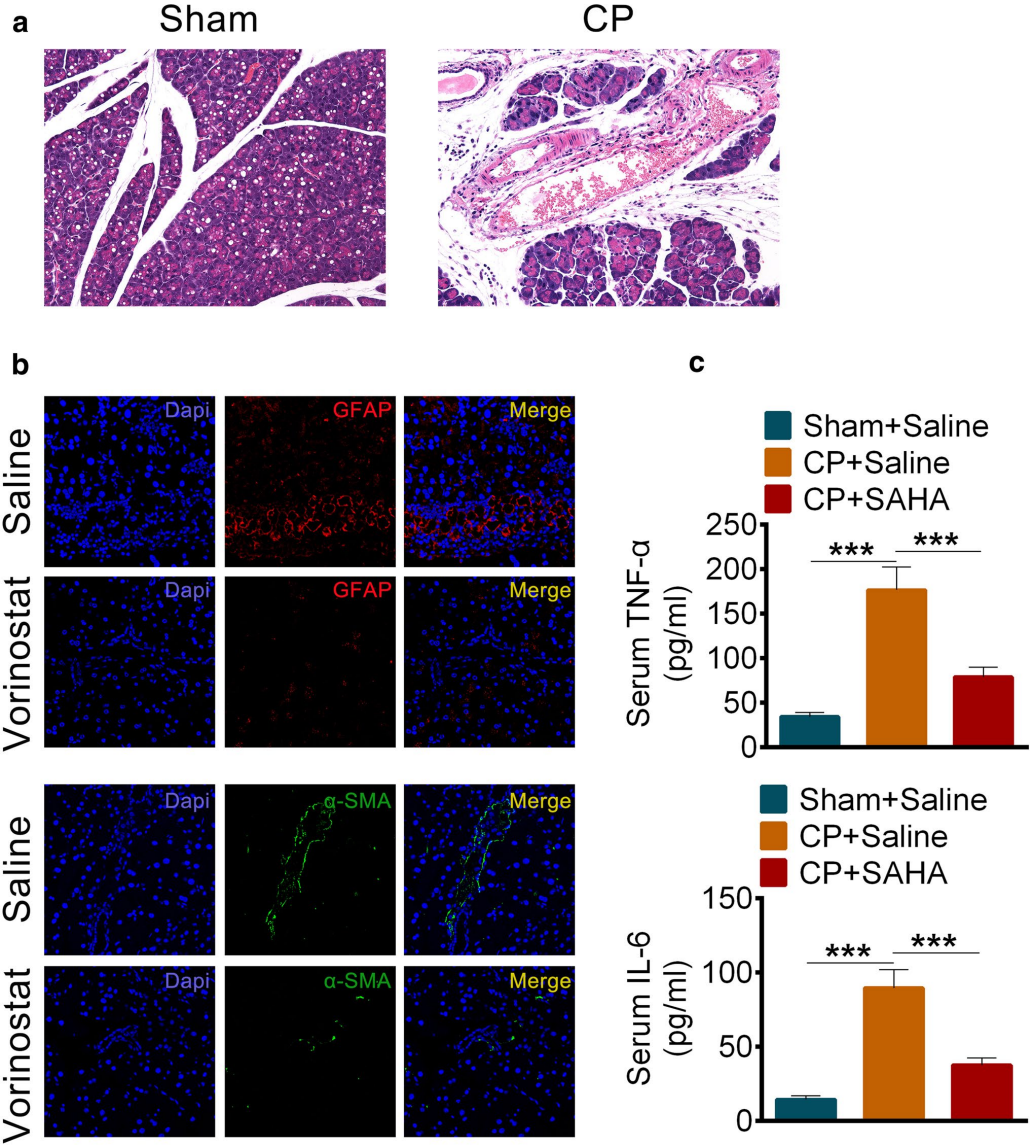
Vorinostat (SAHA) prevented pancreatic fibrosis in rats of chronic pancreatitis. **a** Hematoxylin and eosin dyeing of pancreas sections in chronic pancreatitis. **b** Immunofluorescence staining of GFAP (red, up) or α-SMA (green, down) in pancreatic tissues of rats with chronic pancreatitis after treated with Saline or SAHA (25 mg/kg). **c** The levels of TNF-α (up) and IL-6 (down) in serums of rats with or without chronic pancreatitis after treated with Saline or SAHA (25 mg/kg). All data are presented as the mean ± S.D. (*n* = 5). ***P < 0.001, compared with control. Each assay was performed in triplicate

Rats undergoing repetitive treatment with trinitrobenzene sulfonic acid caused chronic pancreatitis (Fig. 1a). We found that expression of the fibrosis marker GFAP, α-SMA (Fig. 1b) and the inflammation marker TNF-α, IL-6 (Fig. 1c) increased significantly in chronic pancreatitis when compared to controls. However, under the treatment with the HADC inhibitor Vorinostat (also called SAHA), the pancreatic fibrosis phenotypes were rescued remarkably (Fig. 1a, b).

### Vorinostat (SAHA) could restore miR-15 and miR-16 levels in chronic pancreatitis

**Fig. 2:**
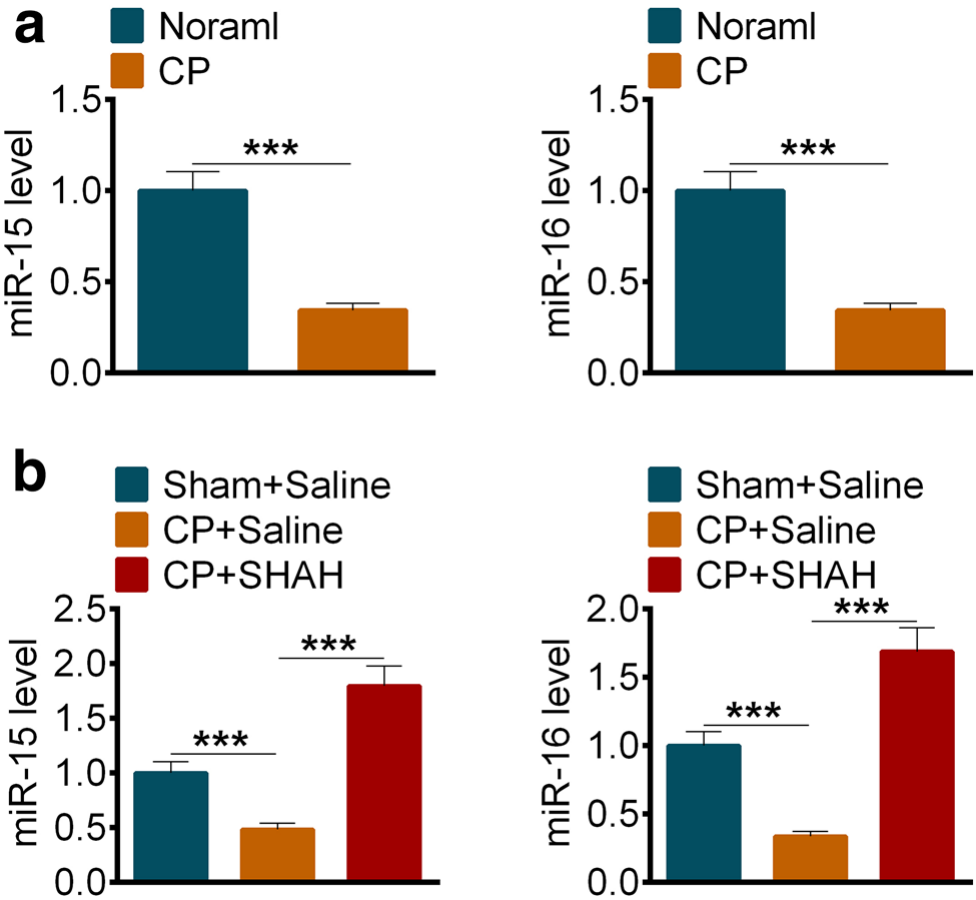
Vorinostat (SAHA) could restore miR-15 and miR-16 levels in chronic pancreatitis. **a** The levels of miR15 (left) and miR-16 (right) in pancreatic tissues of human with or without chronic pancreatitis. (*n* = 10) **b** The levels of miR15 (left) and miR-16 (right) in pancreatic tissues of rats with or without chronic pancreatitis after treated with Saline or SAHA (25 mg/kg). All data are presented as the mean ± S.D. (*n* = 5). ***P < 0.001, compared with control. Each assay was performed in triplicate

According to our results, the levels of miR-15 and miR-16 in chronic pancreatitis tissues of patients strongly decreased (Fig. 2a). We confirmed and extended the studies in our animal chronic pancreatitis models. To our surprise, the amounts of miR-15 and miR-16 rebounded when we treated the rats with the HADC inhibitor Vorinostat (Fig. 2b).

### HDAC inhibition promoted apoptosis of pancreatic stellate cells and elevated transcription of miR-15 and miR-16

**Fig. 3:**
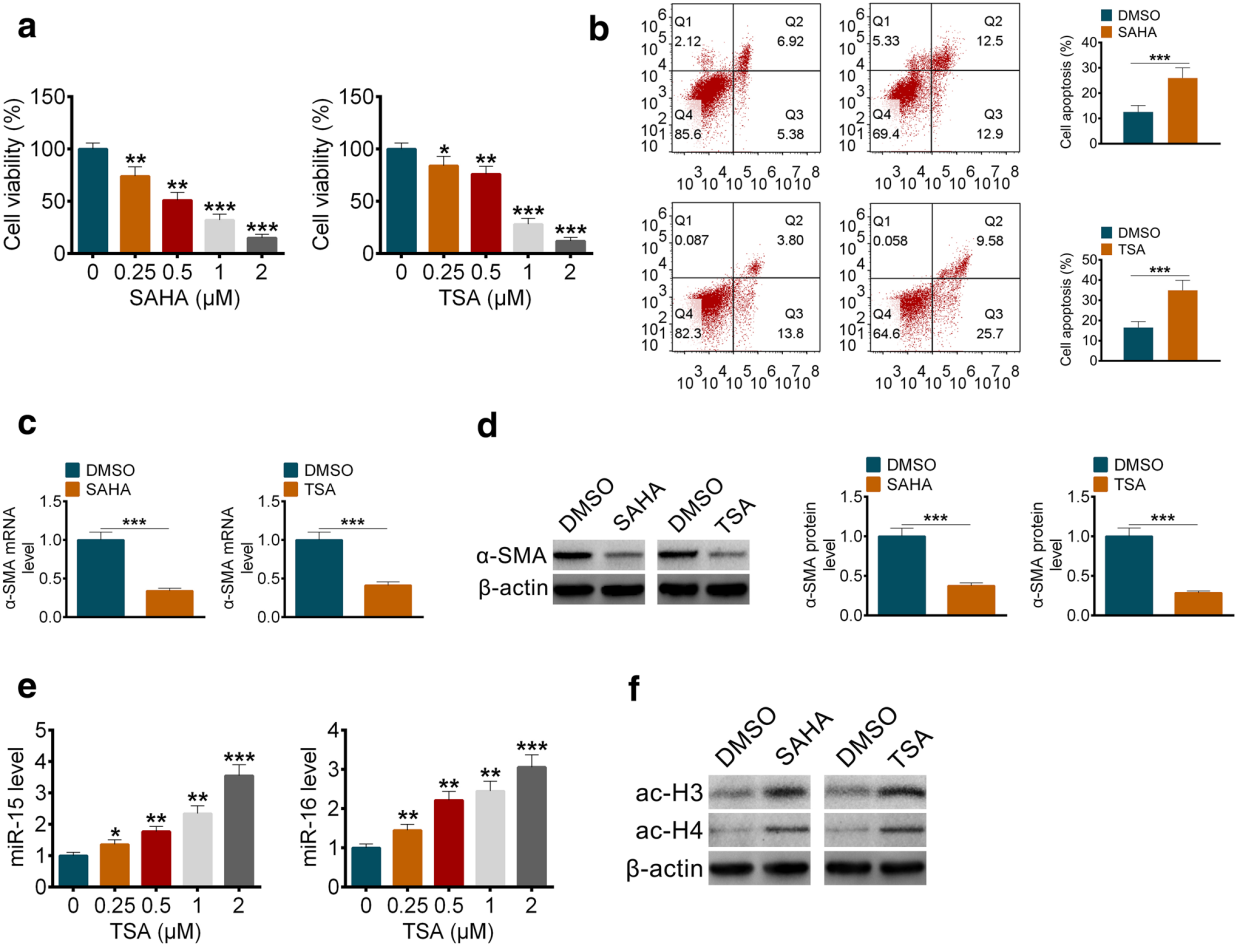
HDAC inhibition promoted apoptosis of pancreatic stellate cells and elevated transcription of miR-15 and miR-16. **a** The cell viability of PSCs treated with an assigned concentration of SAHA (left) or TSA (right). **b** The percentage of apoptosis PSCs treated with SAHA or TSA. **c** The mRNA levels of α-SMA in PSCs treated with SAHA (left) or TSA (right). **d** The protein levels and the related quantification of α-SMA in PSCs treated with SAHA or TSA. **e** The levels of miR15 (left) and miR-16 (right) in PSCs treated with an assigned concentration of TSA. **f** The protein levels of ac-H3 and ac-H4 in PSCs treated with SAHA or TSA. All data are presented as the mean ± S.D. (*n* = 3). ***P < 0.001, compared with control. Each assay was performed in triplicate

To explored how HDAC activities affected the progress of pancreatic fibrosis, we separated the primary pancreatic stellate cells from rats, and the morphology of PSCs showed in Fig S1a, and PSCs was identified by immunofluorescence (Fig S1b, c). As we showed, two different HADC inhibitors, vorinostat (SAHA) or trichostatin A (TSA), dose dependently inhibited the survival of pancreatic stellate cells (Fig. 3a). By FACS, we found HADC inhibitors promoted apoptosis of pancreatic stellate cells indeed (Fig. 3b). And the mRNA (Fig. 3c) and protein (Fig. 3d) levels of α-SMA decreased remarkably when treated with SAHA or TSA, which was consistent with our previous in vitro findings. Interestingly, we found the expression levels of miR-15 and miR-16 in pancreatic stellate cells increased when treated with TSA (Fig. 3e). And the acylation of Histone 3 and Histone 4 was promoted under the incubation of HDAC inhibitors (Fig. 3f), which might induce the transcription of miR-15 and miR-16.

### miR-15 and miR-16 promoted apoptosis of pancreatic stellate cells

**Fig. 4:**
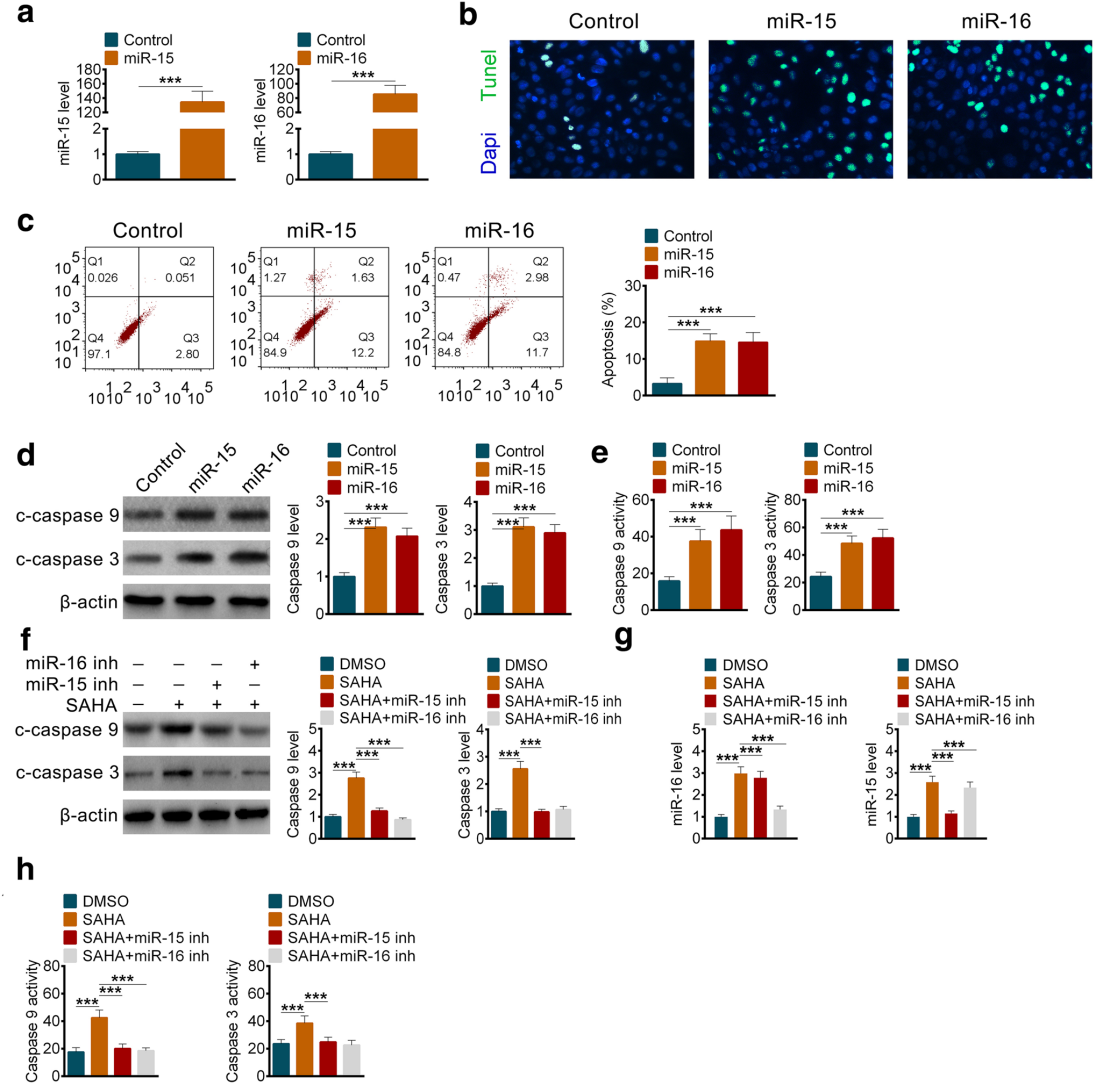
miR-15 and miR-16 promoted apoptosis of pancreatic stellate cells. **a** The expression of miR-15 (left) and miR-16 (right) after overexpression with mimics. **b** Immunofluorescence staining of DNA in PSCs with TUNEL assay after overexpression of miR-15 or miR-16. **c** The percentage of apoptosis PSCs after overexpression of miR-15 or miR-16. **d** The protein levels and the related quantification of cleaved caspase 3 and caspase 9 in PSCs after overexpression of miR-15 or miR-16. **e** The enzyme activities of caspase 3 and caspase 9 in PSCs after overexpression of miR-15 or miR-16. **f** The protein levels and the related quantification of cleaved caspase 3 and caspase 9 in PSCs after transfected with miR-15 inhibitor or miR-16 inhibitor with or without SAHA. **g** The levels of miR15 (left) and miR-16 (right) in PSCs after transfected with miR-15 inhibitor or miR-16 inhibitor. **h** The enzyme activities of caspase 3 and caspase 9 in PSCs after transfected with miR-15 inhibitor or miR-16 inhibitor with or without SAHA. All data are presented as the mean ± S.D. (*n* = 3). ***P < 0.001, compared with control. Each assay was performed in triplicate

Since HDAC inhibition could enhance the transcription of miR-15 and miR-16, then how did they regulate the survival of pancreatic stellate cells. We overexpressed them in pancreatic stellate cells and detected apoptosis of them by TUNEL assay and FACS. The results showed that both miR-15 and miR-16 promoted apoptosis of pancreatic stellate cells indeed (Fig. 4b, c). Moreover, cleaved caspase 9 and cleaved caspase 3 increased obviously when miR-15 and miR-16 were overexpressed (Fig. 4d). And the activities of caspase 9 and caspase 3 improved significantly (Fig. 4e). On the contrary, under the treatment of SAHA, the enhanced activities of caspase 9 and caspase 3 were inhibited by miR-15 inhibitor or miR-16 inhibitor (Fig. 4f, h), meanwhile, qPCR was performed to confirm the efficacy of miR-15 inhibitor or miR-16 inhibitor (Fig. 4g). The above results meant that HDAC inhibition promoted apoptosis of pancreatic stellate cells by miR-15 and miR-16.

### HDAC inhibition disrupts TGF-β/Smad Signaling by miR-15 and miR-16 to protect against pancreatic fibrosis

**Fig. 5:**
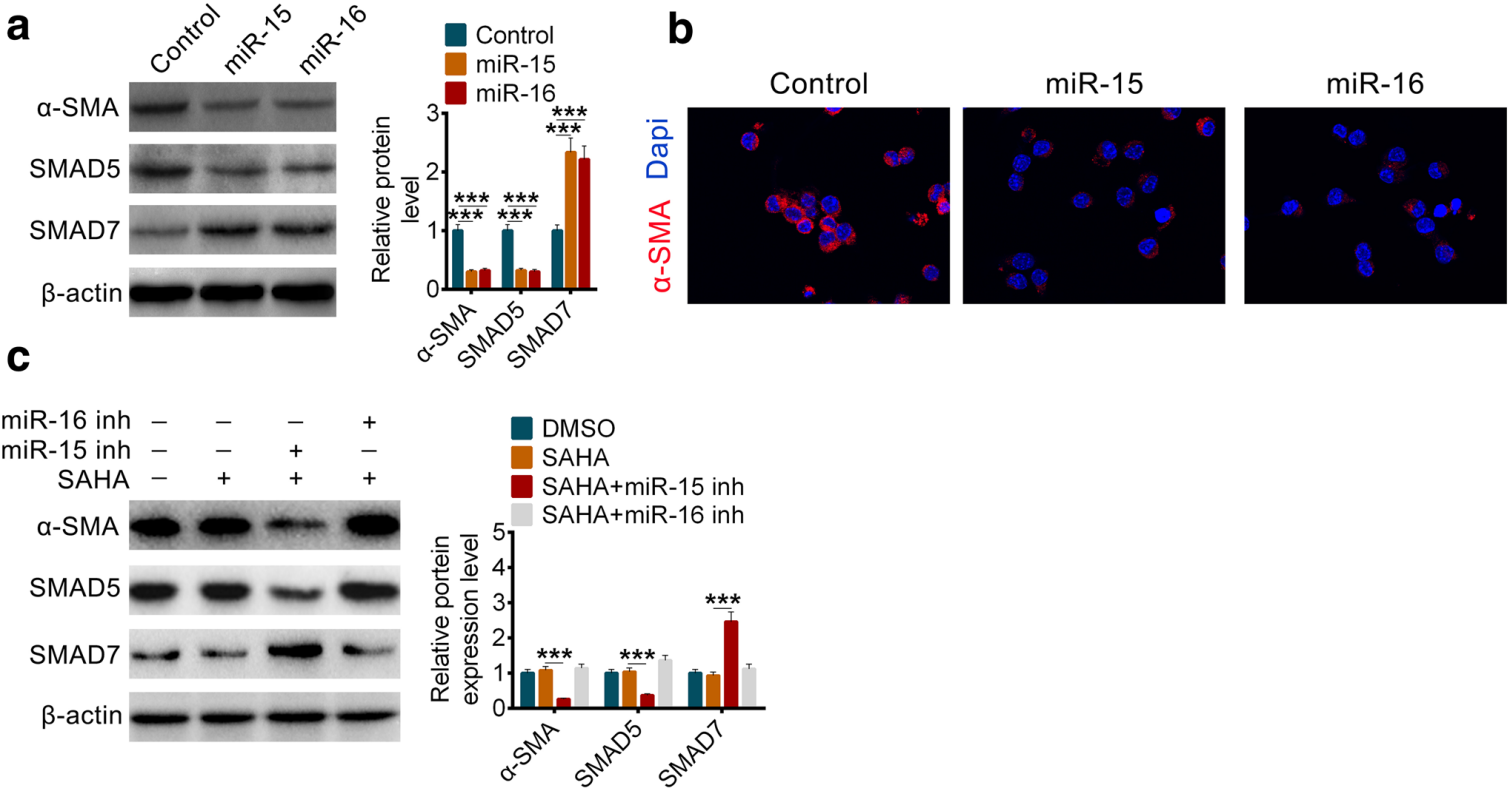
HDAC inhibition disrupts TGF-β/Smad Signaling by miR-15 and miR-16 to protect against pancreatic fibrosis. **a** The protein levels and the related quantification of α-SMA, SMAD5 and SMAD7 in PSCs after overexpression of miR-15 or miR-16. **b** Immunofluorescence staining of α-SMA in PSCs after overexpression of miR-15 or miR-16. **c** The protein levels and the related quantification of α-SMA, SMAD5 and SMAD7 in PSCs after transfected with miR-15 inhibitor or miR-16 inhibitor with or without SAHA. All data are presented as the mean ± S.D. (*n* = 3). ***P < 0.001, compared with control. Each assay was performed in triplicate

Except for the survival of pancreatic stellate cells were regulated by miR-15 and miR-16, we also found that the expression of α-SMA was inhibited at the same time (Fig. 5a, b). Smad5, the major transcription factor of TGF-β, decreased when miR-15 and miR-16 were overexpressed (Fig. 5a). Smad7, another antistatic transcription factor of Smad5, increased as well (Fig. 5a). Similarly, the decreased miR-15 or miR-16 could eliminate the protection of HDAC inhibition to pancreatic fibrosis (Fig. 5c).

### miR-15 and miR-16 induce the apoptosis and fibrosis of pancreatic stellate cells by targeting Bcl-2 and Smad5

**Fig. 6:**
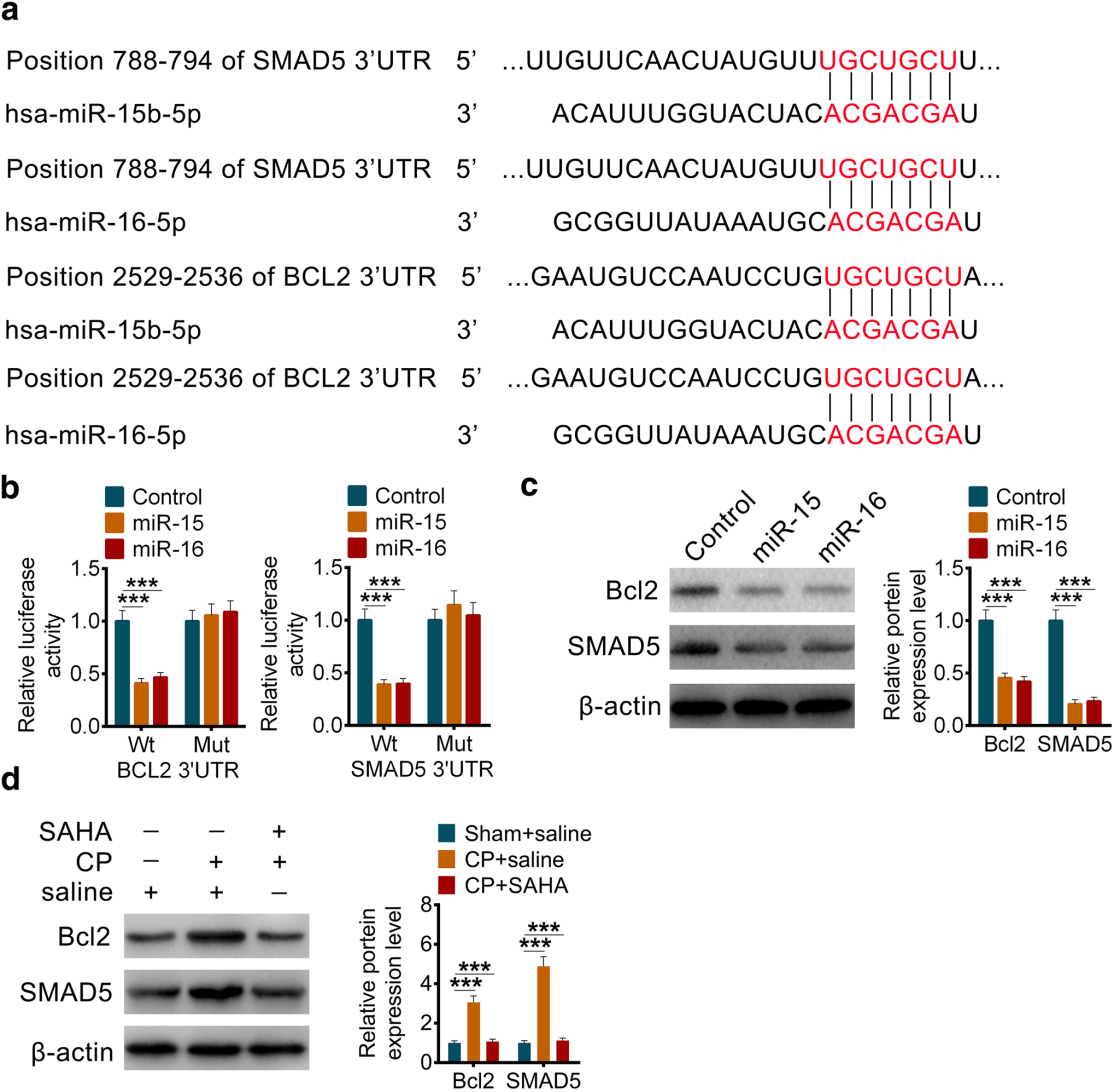
miR-15 and miR-16 induce the apoptosis and fibrosis of pancreatic stellate cells by targeting Bcl-2 and Smad5. **a** miR-15b and miR-16 share conserved binding sites in 3′UTR of Bcl-2 and SMAD5. **b** The luciferase activity of either wildtype (Wt) or mutant (Mut) 3′UTR of Bcl-2 and SMAD5 in PSCs after overexpression of miR-15 or miR-16. **c** The protein levels and the related quantification of Bcl-2 and SMAD5 in PSCs after overexpression of miR-15 or miR-16. **d** The protein levels and the related quantification of Bcl-2 and SMAD5 in pancreatic tissues of rats with or without chronic pancreatitis after treated with Saline or SAHA (25 mg/kg). All data are presented as the mean ± S.D. (*n* = 3). ***P < 0.001, compared with control. Each assay was performed in triplicate

Through the sequence alignment of TargetScan, we found that it was probable that both Bcl-2 and Smad5 were the target genes of miR-15 and miR-16 (Fig. 6a). The reporter gene assays of 3′UTR and their mutant clones showed us miR-15 and miR-16 could bind and regulate the transcription Bcl-2 and Smad5 (Fig. 6b). The decreased protein levels of them further convinced our hypothesis (Fig. 6c). Furthermore, the protein level of Bcl-2 and Smad5 in our animal chronic pancreatitis models were detected, and the result showed that Bcl-2 and Smad5 upregulated in chronic pancreatitis model and decreased while treated with SAHA (Fig. 6d), and negatively correlated with miR-15 or miR-16.

## Discussion

For so long, the relation between acute pancreatitis and chronic pancreatitis has been a great debate. Some evidences revealed that chronic pancreatitis was the result of repeated episodes of acute pancreatitis [37]. And there are four prominent theories of chronic pancreatitis pathogenesis, the toxic-metabolic theory, the oxidative stress hypothesis, the stone and duct obstruction theory and the necrosis-fibrosis hypothesis [1]. Pathological speaking, chronic pancreatitis is a dynamic inflammatory process that is characterized by progressive fibrosis, pain and/or loss of exocrine and endocrine functions [38, 39].

Now Alcohol and some oxidized by-products have been devised to common risk modifiers for chronic pancreatitis [40, 41]. Although the clinical, morphological, and etiological characteristics of chronic pancreatitis are well known, the pathogenic mechanism has remained elusive. In this study, apoptosis and fibrosis transformation regulated by miR-15 and miR-16 were elucidated to help understand the progress of chronic pancreatitis.

Recently studies in vitro and in vivo have shown the vital role of activated PSCs in CP. Following their initial activation, when injury or inflammation are sustained or repeated, the activation of PSCs is retained, assisting the development of pancreatic fibrosis; on the contrary, if the injury or inflammation are suppressed, PSCs may undergo apoptosis or convert to the quiescent stage [42]. HDAC is crucial because DNA expression is regulated by acetylation and de-acetylation of histone during the process of PSCs activation [43]. According to studies, the expression of many miRNAs was changed during PSCs activation, such as miR-324, miR-34c, miR-484, miR-15 and miR-16, leading to the pathological changes like pancreatic fibrosis [30]. Among them, miR-484 would regulate fibrosis by targeting IL-8, while miR-34c could target notch signaling pathway, and miR-324-5p was presented to directly target specificity protein 1 and E26 transformation-specific 1, which played important roles in ECM signaling pathway [30].

Our results show that miR-15 and miR-16 are down-regulated both in human patients and PSCs from rats with CP along with symptoms of fibrosis and inflammation. The application of HDAC inhibitors, SAHA or TSA, demonstrates that miR-15 and miR-16 are regulated by abnormal activation of HADC in PC. And Bcl-2, one of the most important anti-apoptotic proteins, is proved to be a target gene of miR-15 and miR-16. Then the progress of apoptosis is inhibited in CP, and the redundant PSCs with defects will gradually cause some damage. On the other hand, TGF-β is important in many cellular activities, only a few TGF-β activating pathways are already known, and the full mechanism behind this activation pathways is not yet well understood. Activated TGF-β signaling levels could cause several complications including inflammation, autoimmune disorders, fibrosis, cancer and cataracts. Smad5, a kind of receptor-regulated Smads, is a signal transducer for receptors of the TGF-β. And it is able to translocate into the cell nucleus to induce transcription of different effectors then regulate cell development and growth. Coincidentally, Smad5 is also regulated by miR-15 and miR-16 in our study. The results above explained the pathogenesis of CP well in a completely new point of view, which enriches the thoughts for the treatment of CP.

## Electronic supplementary material

Below is the link to the electronic supplementary material.

Supplementary file1. Fig S1. Isolation and identification of PSCs. **a** The morphology of PSCs with cultured 24 h, 48 h and after passage. **b** Immunofluorescence staining of α-SMA in PSCs with cultured 24 h, 48 h and PSCs after passage. **c** Immunofluorescence staining of desmin in PSCs with cultured 24 h, 48 h and PSCs after passage. (JPG 2894 kb)

## Data Availability

All relevant data are within the paper.
