# Supplementary figures and images for: HDAC inhibitors promote pancreatic stellate cell apoptosis and relieve pancreatic fibrosis by upregulating miR-15/16 in chronic pancreatitis
Source: Hum Cell. 2020 Jun 11;33(4):1006–16. doi: 10.1007/s13577-020-00387-x (PMC7505886; doi:10.1007/s13577-020-00387-x)

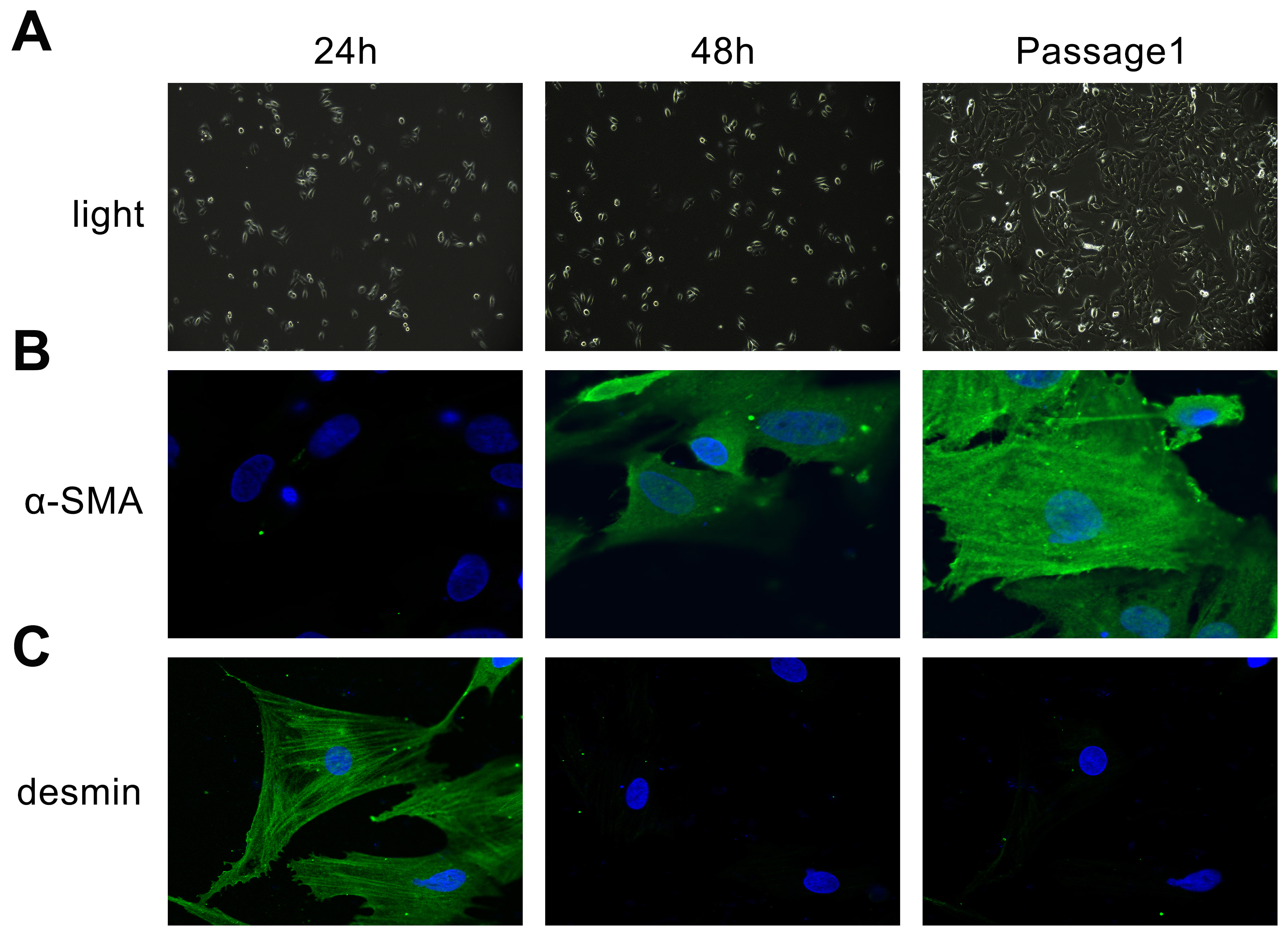

Supplement: Supplementary file 1 — Supplementary file1. Fig S1. Isolation and identification of PSCs. a The morphology of PSCs with cultured 24 h, 48 h and after passage. b Immunofluorescence staining of α-SMA in PSCs with cultured 24 h, 48 h and PSCs after passage. c Immunofluorescence staining of desmin in PSCs with cultured 24 h, 48 h and PSCs after passage. (JPG 2894 kb) [file 13577_2020_387_MOESM1_ESM.jpg]
